# Effective Sequential Combined Chemotherapy with Trifluridine/Tipiracil and Regorafenib in Human Colorectal Cancer Cells

**DOI:** 10.3390/ijms19102915

**Published:** 2018-09-25

**Authors:** Kazuaki Matsuoka, Fumio Nakagawa, Nozomu Tanaka, Hiroyuki Okabe, Kenichi Matsuo, Teiji Takechi

**Affiliations:** 1 Translational Research Laboratory, Taiho Pharmaceutical Co., Ltd., 224-2, Ebisuno Hiraishi, Kawauchi-Cho Tokushima, Tokushima 771-0194, Japan; ttakechi@taiho.co.jp; 2 Applied Pharmacology Laboratory, Taiho Pharmaceutical Co., Ltd., 224-2, Ebisuno Hiraishi, Kawauchi-Cho Tokushima, Tokushima 771-0194, Japan; f-nakagawa@taiho.co.jp; 3 Drug Discovery & Development I Laboratory, Taiho Pharmaceutical Co., Ltd., 3, Okubo, Tsukuba, Ibaraki 300-2611, Japan; noz-tanaka@taiho.co.jp; 4 Product Promotion, Taiho Pharmaceutical Co., Ltd., 1-2-4 Uchikanda, Chiyoda-ku, Tokyo 101-0047, Japan; h-okabe@taiho.co.jp; 5 Pharmacology Laboratory, Taiho Pharmaceutical Co., Ltd., 3, Okubo, Tsukuba, Ibaraki 300-2611, Japan; matsuken@taiho.co.jp

**Keywords:** trifluridine, tipiracil hydrochloride, TAS-102, regorafenib, colorectal cancer

## Abstract

Salvage chemotherapy for refractory metastatic colorectal cancer using trifluridine/tipiracil (FTD/TPI) and regorafenib has shown survival benefits. We evaluated the antitumor effects of FTD or FTD/TPI combined with regorafenib in vitro and in vivo. SW620, HCT 116, and HT-29 human colorectal cancer cell lines were treated with FTD and regorafenib simultaneously and sequentially. Cell death, incorporation of FTD into DNA, and molecules related to FTD and regorafenib-associated cell death were investigated. The antitumor effects of FTD combined with regorafenib in SW620 and COLO205 xenografts were also evaluated. Cell death was greater after sequential treatment with FTD followed by regorafenib in SW620 cells, but not in HCT 116 and HT-29 cells, than after treatment with FTD alone, which was attributable to thymidylate synthase reduction with the induction of apoptosis. In contrast, simultaneous and sequential exposure to regorafenib followed by FTD, but not FTD alone, attenuated the cell death effect. Furthermore, combined FTD/TPI treatment followed by regorafenib had greater antitumor activity than either monotherapy in SW620 and COLO205 xenograft models. Treatment results following regorafenib administration subsequent to FTD or FTD/TPI suggest that sequential therapy with FTD/TPI prior to regorafenib may be effective in a clinical setting.

## 1. Introduction

Colorectal cancer (CRC) is the third most commonly diagnosed cancer worldwide [1]. The management of patients with metastatic CRC (mCRC) requires systemic administration of cytotoxic drugs. Patients with mCRC who receive chemotherapy have a median overall survival (OS) of more than 2 years [1], whereas those receiving standard care alone have a median OS of 5 months [1,2]. Combination therapies using 5-fluorouracil (5-FU)/leucovorin (LV) and oxaliplatin (FOLFOX), as well as 5-FU/LV and irinotecan hydrochloride (FOLFIRI), have been established as effective cytotoxic regimens for the treatment of mCRC [1].

Trifluridine/tipiracil (FTD/TPI, also known as TAS-102) is a combination treatment including FTD and TPI at a molar ratio of 1:0.5. FTD, an antineoplastic thymidine analog [3], is the active antitumor component of FTD/TPI [4,5]. FTD inhibits thymidylate synthase when in its monophosphate form [6] and incorporates into DNA when in its triphosphate form, thereby inhibiting DNA synthesis and resulting in antitumor activity. TPI potently inhibits thymidine phosphorylase [7], an enzyme that degrades FTD. Therefore, TPI helps maintain adequate concentrations of orally administered FTD in the plasma [7], potentiating its antitumor activity. Improvements in OS in patients receiving FTD/TPI treatment were better than those in patients receiving placebo in a global randomized phase III trial (RECOURSE), which included patients with mCRC refractory to standard chemotherapy [8]. FTD/TPI is approved in Japan, the United States, and the European Union, and other countries, for the treatment of unresectable advanced or recurrent colorectal cancer.

Regorafenib is an oral multikinase inhibitor that blocks the activity of several protein kinases associated with angiogenesis, oncogenesis, and the tumor microenvironment [9]. OS was shown to be longer in patients with mCRC refractory to standard chemotherapy receiving regorafenib than in patients receiving a placebo in a randomized phase III trial (CORRECT) [10]. As with FTD/TPI, regorafenib is recognized as a standard treatment for patients with refractory mCRC. Clinical retrospective evaluations have been reported using data from patients who received FTD/TPI or regorafenib in terms of efficacy and safety, suggesting that FTD/TPI and regorafenib have a similar effect on OS but different toxicities [11,12]. However, the administration sequence of these drugs and mechanisms of action in preclinical studies have not been investigated in detail.

In this study, we investigated whether cytotoxicity was enhanced when FTD was used simultaneously or sequentially with regorafenib in vitro. Furthermore, we evaluated the antitumor effects of FTD in combination with regorafenib using in vivo human tumor xenograft models.

## 2. Results

### 2.1. Cell Death after Sequential Administration of FTD Followed by Regorafenib

**Figure 1:**
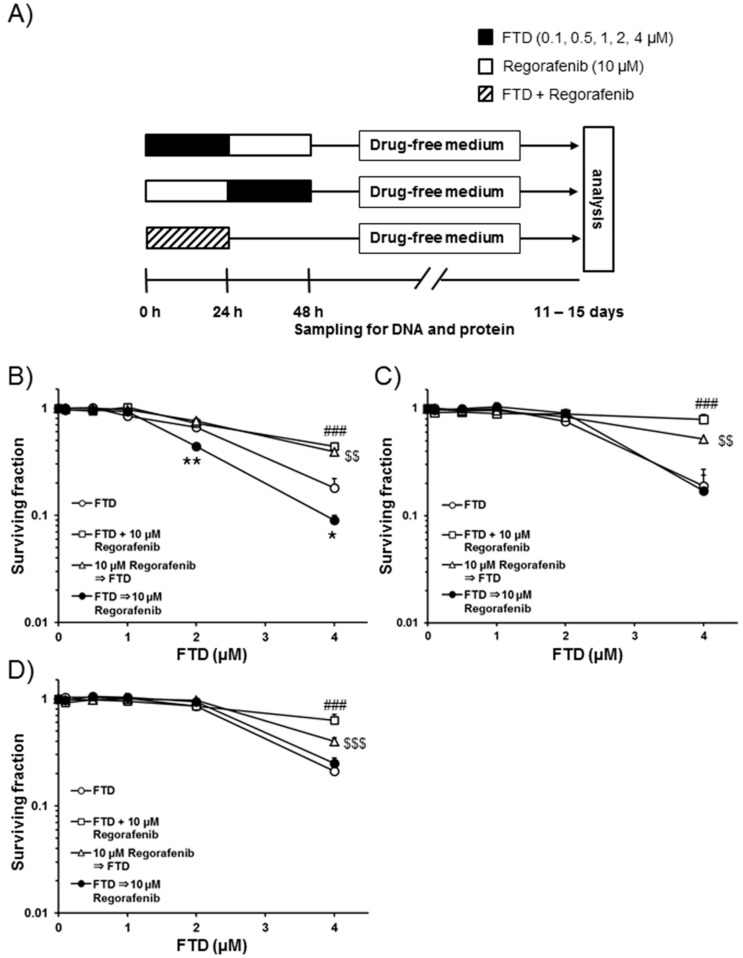
A clonogenic cell survival assay of trifluridine (FTD) and regorafenib using various treatment schedules in SW620, HCT 116, and HT-29 cells. (**A**) Treatment schedules are shown. Cells were plated at appropriate concentrations in duplicate in six-well plates. SW620 (**B**), HCT 116 (**C**), and HT-29 (**D**) cells were exposed to 0.1–4.0 µM FTD alone, in combination with 0.1–4.0 µM FTD and 10.0 µM regorafenib for 24 h, 0.1–4.0 µM FTD for 24 h followed by 10.0 µM regorafenib for 24 h, or 10.0 µM regorafenib for 24 h followed by 0.1–4.0 µM FTD for 24 h. Eleven to fifteen days after removal of the drug, the number of colonies was determined. Data from three independent experiments are presented as the mean + standard deviation (SD); surviving fraction (SF) in 0.1–4.0 µM FTD followed by 10.0 µM regorafenib or regorafenib followed by FTD was calculated by assuming a SF of 1.0 when cells were treated with 10.0 µM regorafenib alone for 24 h. Mean SF values for the sequential combination of 2.0 or 4.0 µM FTD followed by 10.0 µM regorafenib in SW620 cells are shown. * *p* < 0.05 and ** *p* < 0.01 represent significant differences compared with 2.0 or 4.0 µM FTD alone. ### *p* < 0.001, $$ *p* < 0.01, and $$$ *p* < 0.001 represent significant differences compared with 4.0 µM FTD alone.

We evaluated the efficacy of simultaneous treatment with FTD and regorafenib for 24 h in SW620, HCT 116, and HT-29 cells, as well as the sequential combination of the two drugs (FTD for 24 h followed by regorafenib for 24 h or vice versa) using a clonogenic cell survival assay. A fixed concentration of 10.0 µM regorafenib, based on the IC_50_ value for regorafenib alone determined in SW620 cells, was used for this experiment (data not shown). Figure 1A shows the treatment schedule for the two drugs. Figure 1B–D show the results obtained after simultaneous exposure to FTD and regorafenib, the sequential combination of FTD followed by regorafenib, and the sequential combination of regorafenib followed by FTD in SW620, HCT 116, and HT-29 cells, respectively. After simultaneous exposure, the surviving fraction (SF) gradually decreased with increasing concentrations of FTD up to 4.0 µM (Figure 1B–D); the SF values were significantly greater than those in cells treated with FTD alone for all three cell lines (*p* < 0.01). Similar to results from the simultaneous exposure experiment, SF values after sequential exposure to regorafenib followed by FTD were significantly greater than those after exposure to FTD alone in all three cell lines (*p* < 0.05 and 0.01). Further, cell death was observed to a lesser extent after simultaneous treatment with FTD and regorafenib and sequential treatment with regorafenib followed by FTD than after treatment with FTD alone. 

SF values after the sequential combination (FTD followed by regorafenib) treatment were comparable to those after treatment with FTD alone in both HCT 116 and HT-29 cells. In contrast, SF values after sequential exposure to FTD (1.0 and 2.0 µM) followed by regorafenib in SW620 cells were significantly lower (*p* < 0.01 for 2.0 µM FTD and *p* < 0.05 for 4.0 µM FTD) than those after exposure to FTD alone.

### 2.2. Incorporation of Trifluridine (FTD) into Genomic DNA

**Figure 2:**
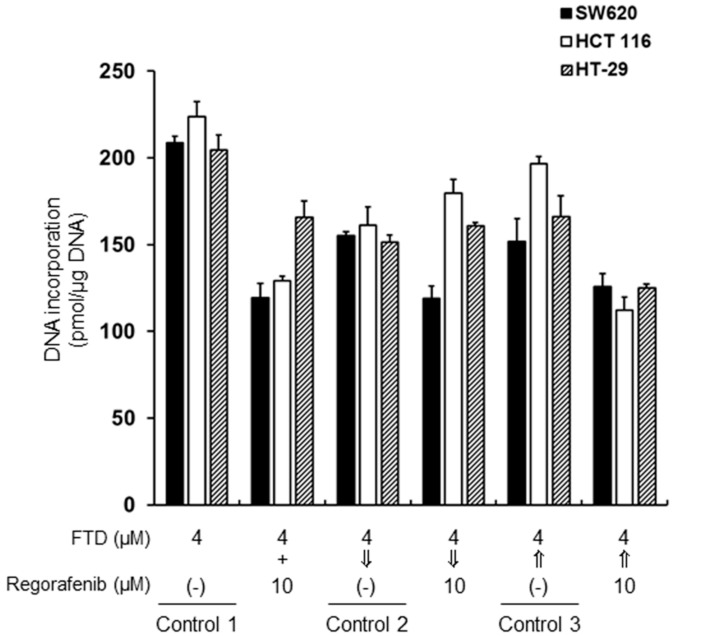
Incorporation of trifluridine (FTD) into DNA. SW620, HCT 116, and HT-29 cells were treated with 4.0 µM FTD and 10.0 µM regorafenib for 24 h, 4.0 µM FTD for 24 h followed by 10.0 µM regorafenib for 24 h, or regorafenib followed by FTD. Double-stranded DNA was extracted, and FTD incorporation was determined via liquid chromatography/tandem mass spectrometry (LC/MS/MS). Data are presented as the mean + SD (*n* = 3).

FTD incorporates into DNA when in its triphosphate form. Therefore, we examined whether incorporation of FTD into DNA differed after simultaneous or sequential exposures to FTD and regorafenib in SW620, HCT 116, and HT-29 cells, as shown in Figure 2. When the cells were simultaneously treated with 4.0 µM FTD and 10.0 µM regorafenib for 24 h, incorporation of FTD into DNA was 60% that of controls (4.0 µM FTD alone for 24 h) in SW620 and HCT 116 cells (for both, *p* < 0.001), and about 80% that of controls in HT-29 cells (*p* < 0.01). Although incorporation of FTD into DNA was significantly less than that observed in control 2 (4.0 µM FTD for 24 h followed by drug-free for 24 h), incorporation of the remaining FTD into DNA was approximately 77% in SW620 cells even after sequential exposure to 4.0 µM FTD for 24 h followed by 10.0 µM regorafenib for 24 h; for comparison, incorporation of the remaining FTD into DNA was approximately 112% and 106% that of control 2 in HCT 116 and HT-29 cells, respectively. In contrast, incorporation of FTD into DNA after sequential exposure to 10.0 µM regorafenib for 24 h followed by 4.0 µM FTD for 24 h was 83%, 57%, and 75% lesser in SW620 (*p* < 0.05), HCT 116 (*p* < 0.001), and HT-29 cells (*p* < 0.01), respectively, than that in control 3 (drug-free for 24 h followed by 4.0 µM FTD for 24 h). These results suggest that regorafenib inhibits the incorporation of FTD into DNA when cells are simultaneously exposed to FTD and regorafenib, as well as during sequential exposure to regorafenib followed by FTD.

### 2.3. Drugs and Apoptosis-Related Protein Expression Following the Combination Treatment

**Figure 3:**
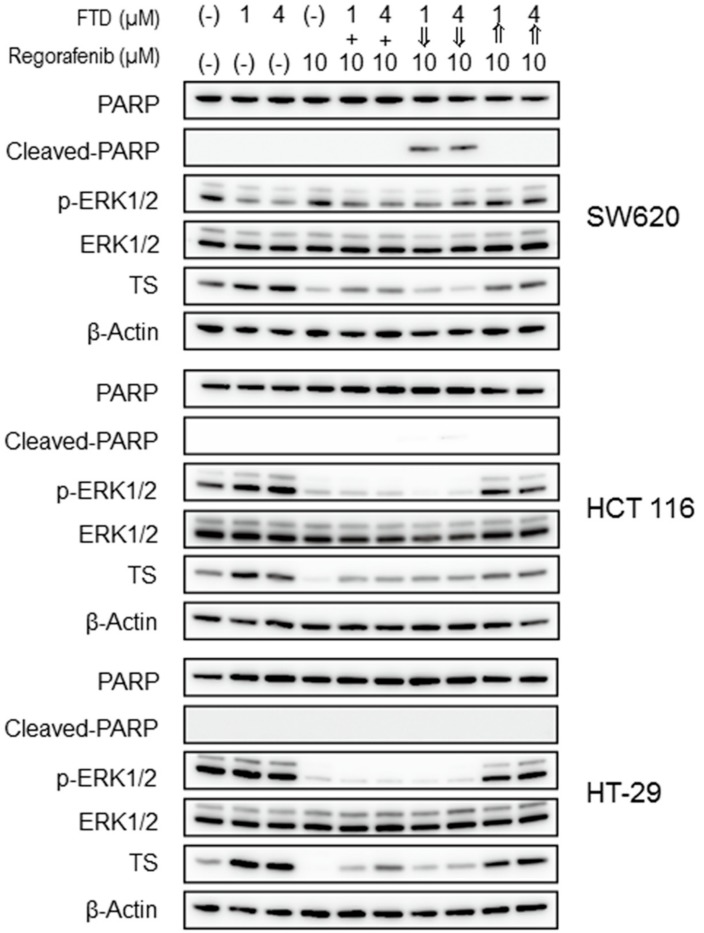
Effects on the expression of phosphorylated extracellular signal-related kinase (p-ERK), ERK, thymidylate synthase (TS), and markers of cell death in SW620, HCT 116, and HT-29 cells using various trifluridine (FTD) and regorafenib treatment schedules. Immunoblots after treatment with 1.0 and 4.0 µM FTD for 24 h, 10.0 µM regorafenib for 24 h, and simultaneous and sequential treatment with 1.0 and 4.0 µM FTD and 10.0 µM regorafenib using the same schedules mentioned above are shown. Definition: PARP—poly(ADP-ribose) polymerase.

We investigated whether drug treatment schedules might influence apoptosis and the expression of phosphorylated extracellular signal-related kinase 1/2 (p-ERK1/2), ERK1/2, and thymidylate synthase (TS); the former and latter are major determinants of the sensitivity of cells to regorafenib and FTD, respectively. The results of the western blot analyses are shown in Figure 3. Similar to a previous report by Schmieder et al. [13], regorafenib alone (10.0 µM) reduced p-ERK1/2 levels in HCT 116 and HT-29 cells, whereas p-ERK1/2 returned to baseline levels in SW620 cells. Consistent with previous results [14,15], FTD alone (1.0 and 4.0 µM) increased p-ERK1/2 levels in HCT 116 cells. In contrast, p-ERK1/2 was not altered in HT-29 cells and was decreased in SW620 cells. Additionally, p-ERK1/2 levels were lower after simultaneous exposure to FTD (1.0 µM or 4.0 µM) and regorafenib (10.0 µM) for 24 h or sequential exposure to FTD (1.0 µM or 4.0 µM) for 24 h followed by regorafenib (10.0 µM) for 24 h than the levels in untreated control cells. A reduction in p-ERK1/2 levels in HCT 116 and HT-29 cells was clearly observed. Conversely, p-ERK1/2 levels in HCT 116 and HT-29 cells recovered after sequential exposure to regorafenib (10.0 µM) for 24 h followed by FTD (1.0 µM or 4.0 µM) exposure for 24 h. No effect on total ERK1/2 expression was observed for all treatment schedules in all three cell lines. 

Treatment with TS inhibitors, such as 5-FU and raltitrexed, rapidly induce TS levels [16], and induction of TS following treatment with FTD alone (1.0 and 4.0 µM) was observed (Figure 3). In contrast, regorafenib reduced TS levels. Especially, the reduction of TS levels in SW620 cells was maintained even after sequential exposure to FTD (1.0 µM or 4.0 µM) for 24 h followed by regorafenib (10.0 µM) for 24 h. In contrast, TS levels were higher after simultaneous exposure to FTD (1.0 µM or 4.0 µM) and regorafenib (10.0 µM) for 24 h and sequential exposure to regorafenib (10.0 µM) for 24 h followed by FTD (1.0 µM or 4.0 µM) for 24 h than those after treatment with regorafenib alone. In both HCT 116 and HT-29 cells, the consistent reductions in TS shown in SW620 cells were not observed for all treatment schedules. Interestingly, cleavage of poly(ADP-ribose) polymerase (PARP), a proapoptotic marker of cellular apoptosis, was dramatically increased in SW620 cells after sequential exposure to FTD (1.0 µM or 4.0 µM) for 24 h followed by regorafenib (10.0 µM) for 24 h.

Collectively, FTD followed by regorafenib treatment reduced TS more than treatment with FTD alone; PARP cleavage, a proapoptotic marker, was also observed in SW620 cells.

### 2.4. Administration of Regorafenib Subsequent to FTD/TPI Increases the Antitumor Effect

**Figure 4:**
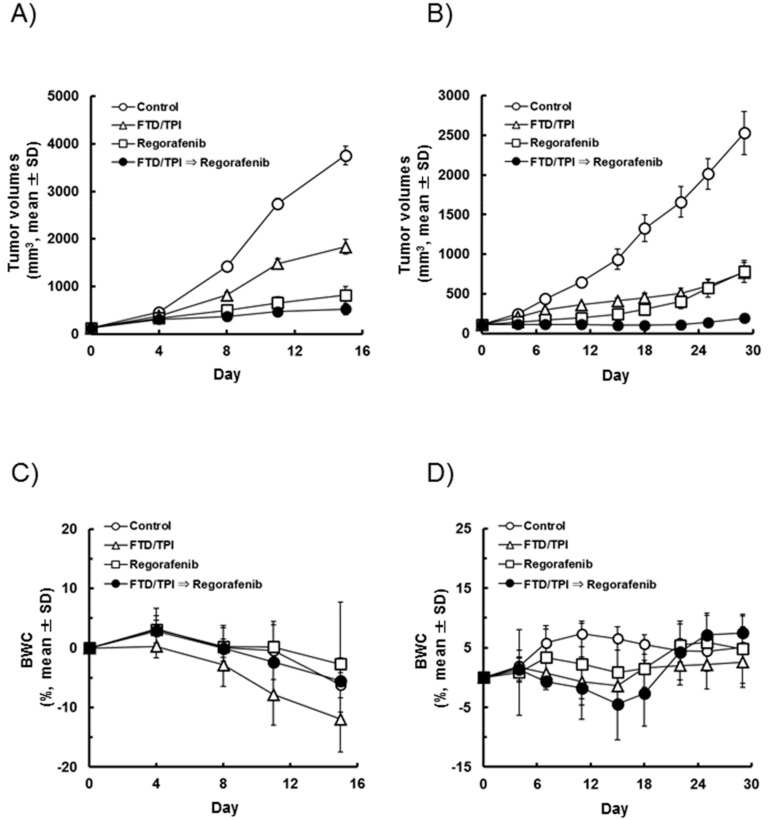
Tumor volume and body weight changes (BWC) in SW620 and COLO205 xenografted mice after daily oral sequential administration of trifluridine/tipiracil (FTD/TPI) and regorafenib. Xenografted mice were randomized on day 0. FTD/TPI (150 mg/kg) and regorafenib (10 mg/kg) were administered orally twice and once daily, respectively, from days 1 to 14. (**A**,**B**) Tumor volume changes and (**C**,**D**) body weight changes in SW620 and COLO205 xenografts, respectively. Values indicate mean ± SD (*n* = 6).

To further evaluate the antitumor effects of a FTD/TPI and regorafenib combination in vivo, we selected SW620 colorectal cells showing significant cell death following treatment using a clonogenic cell survival assay, as shown in Figure 1B. We further selected COLO205 colorectal cells, as the antitumor effects of regorafenib were previously reported in these cells [9]. FTD/TPI (150 mg/kg) and regorafenib (10 mg/kg) either alone or in sequential combination were administered to mice bearing both SW620 and COLO205 colorectal tumors for 14 consecutive days. Tumor volumes and body weight changes (BWC) in SW620 and COLO205 xenografted mice are shown in Figure 4A,B and Figure 4C,D, respectively. Tumor growth was inhibited more in mice treated with FTD/TPI and regorafenib alone than in the controls (*p* < 0.01). Furthermore, the antitumor activity of combined FTD/TPI followed by regorafenib treatment was superior to that of either monotherapy in both SW620 and COLO205 xenografts, as shown in Table 1 and Table 2 (*p* < 0.01). No severe adverse events were observed in the present study, including a greater than 20% reduction in body weight and toxic death. Thus, the combined FTD/TPI followed by regorafenib treatment was well tolerated.

## 3. Discussion

In the present study, we evaluated cell death after simultaneous or sequential combination treatment with FTD and regorafenib in SW620, HCT 116, and HT-29 colorectal cancer cells. Interactions between these drugs in SW620 cells, particularly, were schedule-dependent and more effective when treated sequentially with FTD followed by regorafenib than simultaneous or sequential treatment with regorafenib followed by FTD. These effects were associated with reduced TS expression and apoptosis. Furthermore, we found that sequential combined FTD/TPI followed by regorafenib treatment suppressed tumor growth significantly more than either monotherapy in nude mice xenografted with SW620 or COLO205 colorectal cancer cells, with no significant effect on body weight. Thus, the combination treatment of FTD/TPI and regorafenib exerted significant antitumor activity in an in vivo model.

Initially, we evaluated the simultaneous or sequential treatment of FTD and regorafenib in detail in vitro. Our findings suggest that sequential exposure to FTD followed by regorafenib is more effective in SW620 cells than simultaneous treatments or regorafenib followed by FTD exposures (Figure 1). Surprisingly, cell death was observed to a lesser extent after simultaneous treatment or treatment with regorafenib followed by FTD than after treatment with FTD alone in SW620, HCT 116, and HT-29 cells. To elucidate the mechanisms underlying the attenuated efficacy observed for the combination of FTD and regorafenib, we measured the incorporation of FTD into DNA. Lesser FTD was incorporated into DNA after simultaneous treatment with FTD and regorafenib or sequential treatment with regorafenib followed by FTD than that observed after treatment with FTD alone, resulting in reduced FTD-mediated cell death. FTD is reportedly incorporated into DNA during the S phase [17], and regorafenib strongly induces G0/G1 phase arrest [18]. Therefore, simultaneous or pre-treated regorafenib induces accumulation in the G0/G1 phase and then delays progression to the S phase, which may result in decreased incorporation of FTD into DNA.

In addition, we measured protein expression related to apoptosis and FTD and regorafenib sensitivity to elucidate a cell death mechanism associated with treatments using the combination of FTD and regorafenib. For the first time, we reported a regorafenib-mediated reduction in TS expression. Regorafenib is a sorafenib analog [9], and sorafenib reportedly inhibits E2F-1 expression, resulting in the downregulation of TS expression [19,20]. FTD-induced TS expression occurred via its TS inhibitory activity, as previously reported [5,15]. TS expression after sequential exposure to FTD followed by regorafenib was lower than that after sequential exposure to regorafenib followed by FTD (Figure 3). These results suggest that regorafenib effectively reduces FTD-induced TS expression. In contrast, when cells were post-treated with FTD, TS expression was induced in cells pre-treated with regorafenib. Sorafenib-mediated reductions in TS expression contributed directly to proapoptotic activity [19]; therefore, regorafenib-mediated reductions in TS expression might be the main factor underlying the apoptosis observed after sequential exposure to FTD followed by regorafenib (Figure 3).

Erlotinib and panitumumab suppress FTD-induced ERK phosphorylation, which is the underlying mechanism associated with the combination effects of FTD and erlotinib or panitumumab [14,15]. Reductions in p-ERK1/2 in HCT 116 and HT-29 cells were also observed in this study after simultaneous exposure to FTD and regorafenib, as well as sequential exposure to FTD followed by regorafenib (Figure 3). However, cell death associated with simultaneous and sequential combinatorial treatments was not confirmed in both cell lines (Figure 1). These results suggest that suppression of p-ERK1/2 via regorafenib may not be related to the combination effects of FTD and regorafenib in this study. 

Together, these data suggest that FTD treatment initially increases TS expression, whereas subsequent regorafenib treatment efficiently reduces TS expression, consequently inducing apoptosis in SW620 cells. These results hold only for one of the three cell lines tested; the sequential treatment of FTD followed by regorafenib is more effective in SW620 cells. It remains unclear why the effects of FTD vary depending on the cell line. Further investigations are required to elucidate the mechanisms underlying the effect associated with the order of administration of these drugs using in vivo models.

In conclusion, our findings suggest that the sequential treatment of FTD/TPI followed by regorafenib is significantly more effective than either monotherapy in preclinical models. Moreover, FTD followed by regorafenib is more effective than regorafenib followed by FTD in vitro, suggesting that sequential therapy with FTD/TPI prior to regorafenib may be effective in treating colorectal cancer patients.

## 4. Materials and Methods

### 4.1. Reagents

FTD was obtained from Yuki Gosei Kogyo, Co., Ltd. (Tokyo, Japan), whereas 5-chloro-6-[(2-iminopyrrolidin-1-yl)methyl]pyrimidine-2,4-(1*H*,3*H*)-dione monohydrochloride (tipiracil) was obtained from Taiho Pharmaceutical (Tokyo, Japan). Regorafenib was purchased from Shanghai Haoyuan Chemexpress Co., Ltd. (Shanghai, China), and hydroxypropyl methylcellulose (HPMC) was purchased from Shin-Etsu Chemical (Tokyo, Japan).

### 4.2. Cancer Cell Lines

The SW620 and COLO205 human colon cancer cell lines were purchased from DS Pharma Biomedical Co., Ltd. (Osaka, Japan), and the HCT 116 and HT-29 human colon cancer cell lines were purchased from American Type Culture Collection (ATCC, Manassas, VA, USA). SW620 and COLO205 cells were maintained via implantation into the right axilla of nude mice at 3-week intervals. For in vitro experiments, the cells were cultured at 37 °C in a humidified atmosphere with 95% air and 5% CO_2_. Dulbecco’s modified Eagle medium (DMEM) was used for SW620 and HCT 116 cells and Roswell Park Memorial Institute (RPMI) 1640 medium was used for HT-29 cells, supplemented with 10% fetal bovine serum (Sigma-Aldrich, St. Louis, MO, USA), 100 U/mL penicillin, and 100 µg/mL streptomycin (Nacalai Tesque, Inc., Kyoto, Japan). These cells were authenticated in 2014 by analyzing short tandem repeats.

### 4.3. Animals

Male nude mice were purchased from CLEA Japan (Tokyo, Japan) and were housed under specific pathogen-free conditions, with food and water provided *ad libitum*. All the animal studies were performed according to the guidelines and with the approval of the institutional Animal Care and Use Committee of Taiho Pharmaceutical Co., Ltd. Ethical approval (1 Mar 2013) was obtained prior to conducting the animal experiments.

### 4.4. Clonogenic Cell Survival Assay

The SW620, HCT 116, and HT-29 colorectal cancer cell lines were plated at concentrations of 100–2000 cells/plate in duplicate in six-well plates. Sixteen hours after plating, the cells were treated with FTD and regorafenib as follows: (1) exposure to either 0.1–4.0 µM FTD or 0.4–16.0 µM regorafenib for 24 h, (2) simultaneous exposure to 0.1–4.0 µM FTD and 10.0 µM regorafenib, (3) sequential exposure to 0.1–4.0 µM FTD for 24 h followed by exposure to 10.0 µM regorafenib for 24 h, or (4) sequential exposure to 10.0 µM regorafenib for 24 h followed by exposure to 0.1–4.0 µM FTD for 24 h. Eleven to fifteen days after removal of the drug, cells were fixed with 2% glutaraldehyde and stained with 0.05% crystal violet; subsequently, the number of colonies containing at least 50 cells was determined. Plating efficiency (PE) was calculated by dividing the number of colonies by the number of cells plated. The surviving fractions (SF) for each treatment were determined by normalizing the average PE of each treatment to the PE for 0.1% dimethyl sulfoxide (vehicle).

### 4.5. Determination of FTD Incorporation into DNA

DNA was extracted from cells treated with FTD using NucleoSpin Tissue (Takara Bio, Shiga, Japan), following the manufacturer’s protocol. DNA concentrations were determined using Qubit dsDNA Broad-Range Assay Kits (Thermo Fisher Scientific, Waltham, MA, USA), and samples were diluted to 10 μg/mL with distilled water and degraded to nucleosides using a published method [21]. Incorporation (pmol) of FTD into DNA is presented as the amount of FTD per μg of DNA.

### 4.6. Immunoblotting

Cell pellets were lysed in radioimmunoprecipitation assay (RIPA) buffer (Thermo Fisher Scientific) containing a protease inhibitor cocktail and a phosphatase inhibitor cocktail (Nacalai Tesque, Inc.) and incubated for 30 min on ice. The supernatant was cleared via centrifugation at 15,000× *g* for 15 min at 5 °C. Protein concentrations were determined using a bicinchoninic acid protein assay kit (Thermo Fisher Scientific), and equal amounts of protein (10 µg/lane) were resolved using sodium dodecyl sulfate polyacrylamide gel electrophoresis (SDS-PAGE). An ImageQuant LAS 3000 mini system (GE Healthcare UK Ltd., Buckinghamshire, UK) was used to detect the proteins. The following antibodies were purchased from Cell Signaling Technology (Beverly, MA, USA): anti-PARP (9542S), anti-cleaved PARP (5625S), anti-phospho ERK1/2 (9101S), and anti-ERK1/2 (4696S). Anti-thymidylate synthase (TS) was produced in Taiho Pharm. Co., Ltd., (Tokyo, Japan), and anti-β-actin (clone AC-74) was purchased from Sigma-Aldrich. 

### 4.7. Antitumor Activity In Vivo

The animals were quarantined for one week and then subcutaneously implanted with a solid human tumor of approximately 8 mm^3^ [22]. In order to evaluate the antitumor activity, the mice were randomized on day 0 based on tumor volume, once the mean tumor volume had reached about 100–300 mm^3^. Each treatment group consisted of six mice. FTD/TPI was prepared by mixing FTD and TPI at a molar ratio of 1:0.5 in 0.5% HPMC solution. The dose of FTD/TPI, expressed according to the amount of FTD, was administered orally twice daily from day 1 to 14 at approximately six-hour intervals at the reported effective dose (150 mg/kg/day) [23,24]. For the control group, vehicle (0.5% HPMC solution) was administered at 10 mL/kg in a similar manner. Regorafenib was prepared in polyethylene glycol 400 and aqueous methanesulfonic acid (125 mM, 80:20 *v*/*v*) according to a previous report [9], and was administered orally from day 1 to 14 once a day 2–4 h after the first FTD/TPI administration at the reported effective dose (10 mg/kg/day) [9].

Tumor diameters were measured twice a week, and tumor volume was estimated as 0.5 × length × width^2^. Relative tumor volume (RTV) was calculated using the following formula:

RTV = (tumor volume on measured day)/(tumor volume on day 0). (1)

On day 15 or 29, the tumor growth inhibition ratio (TGI, %) was calculated using the following formula:

TGI (%) = [1 − (RTV in experimental group)/(RTV in control group)] × 100 (%). (2)

Groups were terminated after tumors had reached the ethically allowed limits. Finally, toxicity was evaluated based on BWC, which was calculated using the following formula:

BWC (%) = [(body weight on measured day − body weight on day 0)/body weight on day 0] × 100 (%). (3)

Toxicity was defined as a BWC loss of more than 20% or toxic death. The experimental endpoint was defined as the day on which the average tumor volume for the average body weight within each group reached more than 10%.

### 4.8. Statistical Analysis

Significant differences in the clonogenic cell survival assay were analyzed using the student’s *t*-test. Significant differences in the mean RTV between the treated and the control groups on day 15 or 29 were analyzed using the Aspin-Welch two-sided *t*-test. The combinational antitumor effect of FTD/TPI and regorafenib was analyzed according to a closed testing procedure using the Aspin-Welch two-tailed *t*-test [25]. Differences with an associated *p* value of less than 0.05 were considered significant. *P* values were calculated using EXSUS (ver. 8.1 CAC EXCARE Corp., Osaka, Japan).

## Figures and Tables

**Table 1:** Anti-tumor effects of trifluridine/tipiracil (FTD/TPI) and regorafenib in mice implanted with SW620 human colorectal tumors.

Group	Dose (mg/kg/day)	Treatment	*n*	RTV(mean ± SD)	TGI(%)
Control	-	-	6	29.94 ± 3.40	-
FTD/TPI	150	Day 1–14 (b.i.d.)	6	14.57 ± 1.44 ^a^	51.3
Regorafenib	10	Day 1–14 (q.d.)	6	6.55 ± 1.34 ^a^	78.1
FTD/TPI followed by Regorafenib	150 + 10	-	6	4.07 ± 0.56 ^a,b^	86.4

RTV: Relative tumor volume on day 15; TGI: Tumor growth-inhibition ratio on day 15; b.i.d.: bis in die; q.d.: quaque die; ^a^ *p* < 0.01 with a two-sided Aspin-Welch *t*-test, compared to control; ^b^ *p* < 0.01 with a two-sided Aspin-Welch *t*-test, compared to either monotherapy.

**Table 2:** Anti-tumor effects of trifluridine/tipiracil (FTD/TPI) and regorafenib in mice implanted with COLO205 human colorectal tumors.

Group	Dose (mg/kg/day)	Treatment	*n*	RTV(mean ± SD)	TGI(%)
Control	-	-	6	22.83 ± 2.07	-
FTD/TPI	150	Day 1–14 (b.i.d.)	6	7.02 ± 1.02 ^a^	69.3
Regorafenib	10	Day 1–14 (q.d.)	6	7.09 ± 1.05 ^a^	68.9
FTD/TPI followed by Regorafenib	150 + 10	-	6	1.76 ± 0.29 ^a,b^	92.3

RTV: Relative tumor volume on day 29; TGI: Tumor growth-inhibition ratio on day 29; b.i.d.: bis in die; q.d.: quaque die; ^a^ *p* < 0.01 with a two-sided Aspin-Welch *t*-test, compared to control; ^b^ *p* < 0.01 with a two-sided Aspin-Welch *t*-test, compared to either monotherapy.

## Abbreviations

BWC	Body weight change
CRC	Colorectal cancer
DMEM	Dulbecco’s modified Eagle medium
ERK	Extracellular signal-related kinase
FBS	Fetal bovine serum
FTD	Trifluridine
LV	Leucovorin
PARP	Poly(ADP-ribose) polymerase
PE	Plating efficiency
p-ERK1/2	Phosphorylated extracellular signal-related kinase 1/2
RIPA	Radioimmunoprecipitation assay
RTV	Relative tumor volume
SDS-PAGE	Sodium dodecyl sulfate polyacrylamide gel electrophoresis
SF	Surviving fractions
TGI	Tumor growth inhibition
TS	Thymidylate synthase
TPI	Tipiracil hydrochloride
5-FU	5-Fluorouracil
